# Performance, metabolic and hormonal responses of grazing Nellore cows to an energy-protein supplementation during the pre-partum phase

**DOI:** 10.1186/s12917-020-02309-3

**Published:** 2020-04-09

**Authors:** Matheus Fellipe de Lana Ferreira, Luciana Navajas Rennó, Edenio Detmann, Mário Fonseca Paulino, Sebastião de Campos Valadares Filho, Samira Silveira Moreira, Hudson Caio Martins, Bruno Inácio Correa de Oliveira, Julia Avansi Marquez, Isabela de Paula Cidrine

**Affiliations:** 1 Universidade Federal de Viçosa, Viçosa, Minas Gerais 36570–000 Brazil; 2 Animal Science Department, Federal University of Viçosa, Peter Henry Rolfs Avenue, Viçosa, MG, CEP: 36570-900 Brazil; 3 Universidade de São Paulo, Pirassununga, São Paulo, 13635-900 Brazil; 4 Pontifícia Universidade Católica do Paraná, Curitiba, Paraná 80215-90 Brazil

**Keywords:** Nutrition, Metabolism, Parturition, Zebu

## Abstract

**Background:**

Supplementation programs for prepartum beef cows are usually adopted because the nutritional status of the cows upon calving is a main factor impacting reproductive performance of the next production cycle. This study evaluated the effects of 60-d pre-partum energy-protein supplementation on performance, metabolic and hormonal responses during the peripartum phase of grazing beef cows. Thirty-eight pregnant multiparous Nellore cows were assigned to a completely randomized design with two treatments: control (no supplement) and supplementation (1.5 kg of energy-protein per d with 30% crude protein, dry matter basis).

**Results:**

The supplemented cows had higher ADG pre-partum (*P* < 0.10), but postpartum ADG did not differ between treatments. Supplementation did not affect BCS and calf BW upon calving, on days 45 and 90, milk yield and composition (*P* > 0.10). No differences were found for forage intake and neutral detergent fiber digestibility (P > 0.10). The intake and digestibility of CP and OM increased in response to supplementation (*P* < 0.10). An interaction occurred between supplementation and peripartum days for BUN, βHB, T3 and T4, which had higher concentrations for supplemented cows at pre-partum period (P < 0.10). Concentration of others blood parameters significantly changed along peripartum days (P < 0.10). There was no difference in pregnancy rates and days from calving to conception between treatments (*P* > 0.10).

**Conclusions:**

Providing an energy and protein supplement to grazing Nellore cows over the last 60 d of gestation improved their pre-partum energy balance. However, no post-partum carryover effects were detected.

## Background

The nutritional status upon calving is the main factor influencing the duration between calving and the next conception [1, 2]. It affects reproductive performance and, consequently, the economic success of any beef cattle operation. Inadequate dietary energy supply during late gestation impairs reproduction, even when it is sufficient during lactation. Hence, there is clear evidence that supplementation of beef cows pre-partum is more important than postpartum [3, 4].

In the tropics, beef cows typically spend most of their gestation period during the dry season, which is characterized by low forage yield and quality. Therefore, supplementation of additional energy and protein during the dry season can be an effective measure to improve the tropical conditions [5, 6].

It has been recently shown that a supplementation period over the last 60 d prepartum decreases the negative energy balance during the postpartum phase and reduces the number of days from calving to conception [7]. However, other experiments do not corroborate this [8, 9] and show inconsistent effects of late gestation supplementation on performance and metabolic parameters.

Changes in blood hormones and metabolite concentrations during the peripartum may be interpreted as metabolic cues that relate nutrition to physiology [10], and may help to accurately indicate the effects of supplementation on animal metabolism. The aim of this study was to evaluate the effects of a supplementation program over 60 d pre-partum on the peripartum performance, metabolic and hormonal responses of grazing Nellore cows.

## Results

The average dry matter (DM) and potentially digestible DM (pdDM) yield during the experiment were 2.74 t/ha and 1.70 t/ha.

**Fig. 1:**
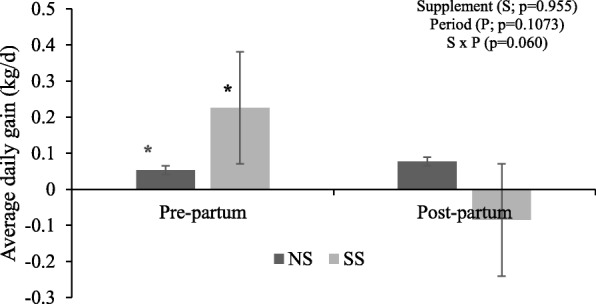
Average daily gain during pre- and post-partum period. Asterisks (*) indicate significant differences between treatments (*P* < 0.10). NS: Nonsupplemented cows, SS: Supplemented cows

An interaction occurred between treatment and period (pre-partum and postpartum) for ADG (*P* < 0.10). The SS cows had higher ADG during the pre-partum (P < 0.10), but ADG did not differ between treatments during the postpartum period. ADG in the NS cows did not vary among periods (*P* > 0.10; Fig. 1).

Supplementation did not affect calving BW, calving BCS and 45-d post-partum, CBW and calf weight at 45- and 90-d, days from parturition to conception, pregnancy rate (Table 1), milk yield and composition on d 30 and 45 of lactation (*P* > 0.10; Table 2). Total DM, organic matter (OM), crude protein (CP) and digestible OM intake were higher (*P* < 0.10) for the supplemented cows, but forage (DMF), apNDF and iNDF intake were not affected by treatments (*P* > 0.10; Table 3). However, when expressed in g/kg BW, the above-cited intakes were not influenced by supplement supply (*P* > 0.10).

**Table 1:** Least square means and *P*-values for effect of energy-protein supplementation on cows and calves’ performance

Item^1^	Treatment		SEM	*P*-value
	NS	SS		
Calving BW (kg)	515.1	536.9	13.86	0.315
Calving BCS	5.19	5.53	0.222	0.233
BCS 45	5.38	5.11	0.273	0.492
CBW (kg)	30.8	32.1	1.14	0.442
CBW45 (kg)	70.6	73.0	1.78	0.380
CBW90 (kg)	93,2	101.1	6.30	0.409
N° days to conception	80	77	11.30	0.874
Pregnancy rate (%)	73	68	–	0.720

^1^*BW* body weight, *BCS* Body Condition Score, *CBW* Calf Birth Weight, *CBW45* Calf Body Weight at 45-d, *CBW90* Calf Body Weight at 90-d

**Table 2:** Least square means and *P*-values for effect of energy-protein supplementation on milk production and composition

Item^1^	Treatment		SEM	*P*-value
	NS	SS		
Milk 30 (kg)	7.69	7.84	0.432	0.817
FCM ^a^ 30 (kg)	8.68	8.43	0.521	0.742
Fat (%)	5.00	4.90	0.205	0.755
Protein (%)	2.99	3.02	0.054	0.697
Lactose (%)	4.60	4.65	0.066	0.557
Total solids (%)	13.40	13.31	0.285	0.825
Milk 45 (kg)	7.74	8.15	0.424	0.525
FCM ^a^ 45 (kg)	9.17	9.41	0.449	0.717
Fat (%)	5.40	5.17	0.244	0.514
Protein (%)	3.05	3.09	0.057	0.598
Lactose (%)	4.63	4.65	0.047	0.673
Total solids (%)	13.89	14.07	0.221	0.556

^a^FMC = 4% fat-corrected milk yield (30 and 45-d)

**Table 3:** Least square means and *P*-values for effect of energy-protein supplementation on cow’s intake during pre-calving

Item	Treatments			SEM	*P-*value
	NS		SS		
		kg/d			
DM	7.82		8.88	0.317	0.056
DMF	7.82		7.54	0.317	0.555
OM	7.29		8.29	0.291	0.052
CP	0.38		0.80	0.015	< 0.001
apNDF	5.96		5.86	0.237	0.775
iNDF	2.66		2.57	0.105	0.569
dOM	2.93		3.91	0.227	0.018
dNDF	3.02		2.94	0.171	0.751
		g/kg BW			
DM	15.33		17.24	0.742	0.118
DMF	15.33		14.63	0.718	0.518
OM	14.31		16.09	0.684	0.114
apNDF	11.68		11.39	0.539	0.701
iNDF	5.22		4.99	0.236	0.523

*DM* Total dry matter intake, *DMF* dry matter of forage intake, *OM* organic matter, *CP* crude protein, *apNDF* neutral detergent fibre corrected for ash and protein, *iNDF* indigestible NDF, *dOM* digested organic matter, *dNDF* disgested NDF

Pre-partum supplementation improved the digestibility of OM and CP (*P* < 0.10). It had no effect on apNDF digestibility or NMic, but affected microbial protein synthesis efficiency (Emic, g CP/kg dOM), which was higher for the NS animals (P > 0.10; Table 4).

**Table 4:** Least square means and P-values for effect of energey-protein supplementation on apparent digestibility and synthesis of nitrogen compounds during pre-calving

Item	Treatments			*P-*value
	NS	SS	SEM	Sup
OM	39.86	47.32	1.522	0.013
CP	2.77	50.65	1.623	< 0.001
apNDF	50.67	50.21	0.014	0.832
Nmic	89.24	92.69	66.535	0.370
Emic	190.06	148.16	12.666	0.036

Organic matter (OM, %), crude protein (CP, %), neutral detergent fiber corrected for ash and protein (apNDF, %), digested organic matter (dOM, g/kg DM), ruminal synthesis of microbial nitrogen (NMic, g/d), efficiency for synthesis of microbial protein (Emic, g microbial CP synthesis kg dOM intake)

No interaction was observed between supplement and peripartum days for blood glucose concentration, triglycerides, total cholesterol, HDL, LDL and VLDL (P > 0.10; Table 5) and supplementation did not affect these variables (P > 0.10). However, the concentration of these variables changed significantly during peripartum days (*P* < 0.10).

**Table 5:** Least square means and P-values for effect of supplementation on serum metabolites and hormones during pre and post-calving

Item	Treatments			*P-*value		
	NS	SS	SEM	Sup^2^	Day^3^	Sup x Day
Glucose, mg/dL	62.97	63.70	1.895	0.793	< 0.001	0.124
Triglycerides, mg/dL	26.74	27.07	1.085	0.835	< 0.001	0.316
Total cholesterol, mg/dL	132.04	142.47	5.385	0.219	< 0.001	0.458
VLDL mg/ dL,	5.34	5.41	5.385	0.219	< 0.001	0.458
LDL mg/ dL,	56.21	66.15	5.054	0.213	< 0.001	0.558
HDL mg/ dL,	69.45	70.58	3.339	0.819	< 0.001	0.309
Creatinine, mg/dL	1.40	1.39	0.054	0.754	< 0.001	0.289
BUN, mg/dL	14.46	15.18	0.836	0.562	< 0.001	< 0.001
Total Proteins, g/dL	7.39	7.43	0.124	0.821	< 0.001	0.436
Albumin, g/dL	3.26	3.24	0.042	0.723	< 0.001	0.735
Globulins, g/dL	4.15	4.19	0.149	0.867	< 0.001	0.453
NEFA, mmol/L^1^	0.33	0.27	0.042	0.377	< 0.001	0.206
βHB, mmol/L^1^	0.47	0.45	0.021	0.676	0.073	0.026
IGF-1, ng/dL	184.64	196.54	16.670	0.629	< 0.002	0.360
Insulin, μIU/mL	2.99	2.83	0.324	0.737	0.0019	0.806
T3, ng/mL	0.637	0.823	0.1305	0.350	0.006	0.025
T4, μg/dL	4.66	5.81	0.689	0.282	<.001	<.001
Progesterone, ng/mL,	0.87	1.02	0.2461	0.687	0.0008	0.674

^1^*NEFA* Non-esterified fatty acids, *βHB* β-hydroxybutyrate

^2^/ Supplementation (Sup)

^3^/ Day relative to calving (Day)

**Fig. 2:**
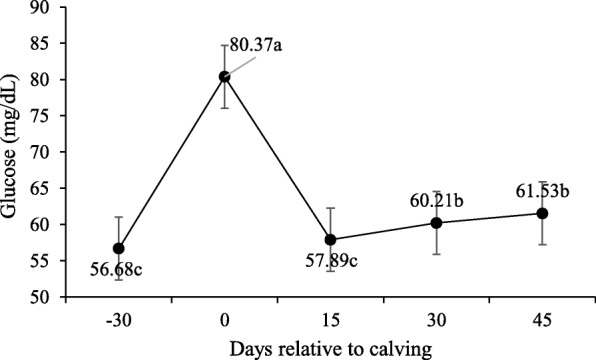
Glucose plasma concentrations during pre- and post-calving. Different letters indicate significant differences between days (*P* < 0.10)

**Fig. 3:**
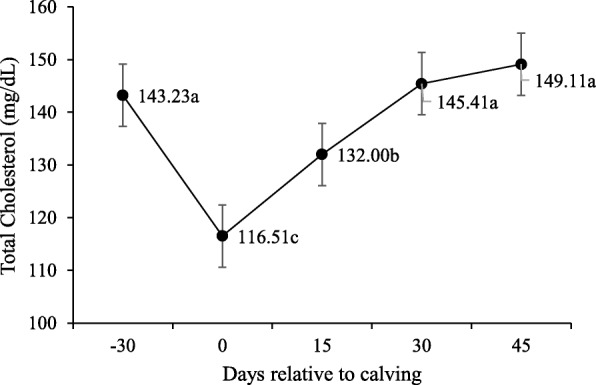
Total cholesterol serum concentrations during pre- and post-calving. Different letters indicate significant differences between days (*P* < 0.10)

**Fig. 4:**
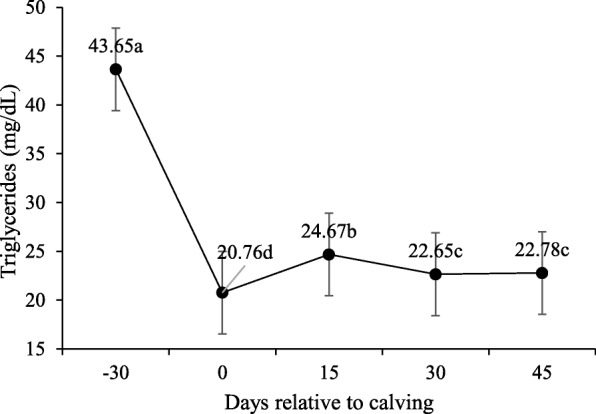
Triglycerides serum concentrations during pre- and post-calving. Different letters indicate significant differences between days (*P* < 0.10)

**Fig. 5:**
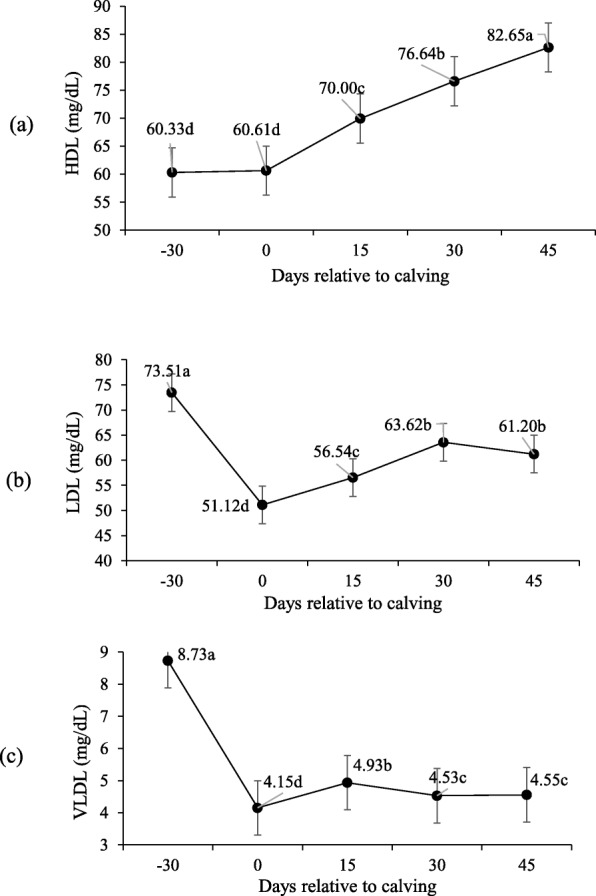
HDL (**a**), LDL (**b**) and VLDL (**c**) serum concentrations during pre- and post-calving. Different letters indicate significant differences between days (*P* < 0.10)

For glucose (Fig. 2), higher serum concentrations were observed upon calving (day 0–80,37 mg/dL), before lowering at 15-d and then stabilizing at the baseline (P < 0.10). Lower serum concentrations were observed for total cholesterol (*P* < 0.10; Fig. 3) and LDL upon calving, which then increased from calving to day 30. Higher HDL concentrations were recorded on day 45. Conversely, triglycerides and VLDL serum levels were higher on − 30-d than for calving and post-partum (Figs. 4 and 5).

Supplementation did not affect the serum concentration of total proteins, albumin, globulins and creatinine during both pre- and post-partum, and only were different during peripartum days (*P* > 0.10; Table 5).

**Fig. 6:**
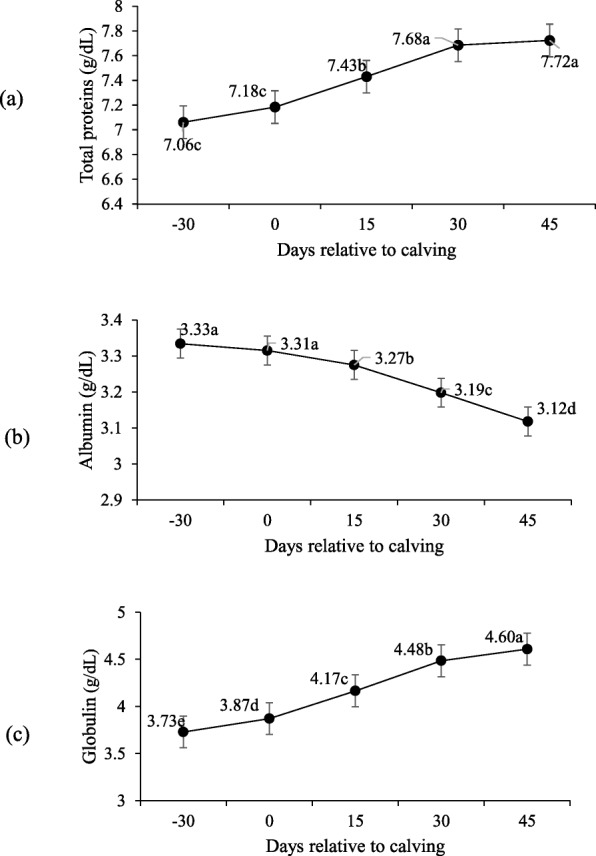
Total protein (**a**), albumin (**b**) and globulin (**c**) serum concentrations during pre- and post-calving. Different letters indicate significant differences between days (*P* < 0.10)

**Fig. 7:**
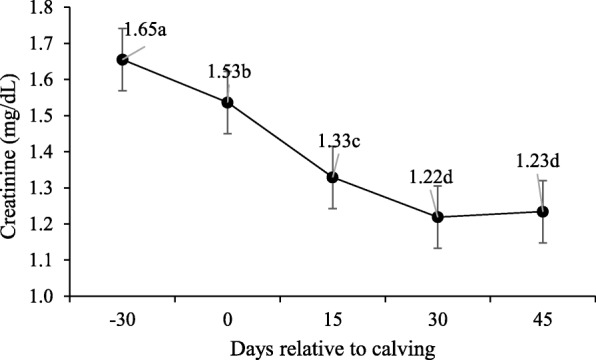
Creatinine serum concentrations during pre- and post-calving. Different letters indicate significant differences between days (*P* < 0.10)

Total proteins in the serum concentrations were similar on days 30 and 45 (*P* > 0.10; Fig. 6a), and higher compared to day − 30, 0 and 15 (*P* < 0.10). Similar serum concentrations were observed for serum albumin (Fig. 6b) on day − 30 and upon calving (P > 0.10), but were higher than on days 15, 30 and 45 (*P* < 0.10). Higher serum concentrations were found for globulins (Fig. 6c) on day 45 compared to the rest of the period. Creatinine lowered throughout the peripartum, with the lowest values noted on days 30 and 45 postpartum (*P* < 0.10; Fig. 7).

**Fig. 8:**
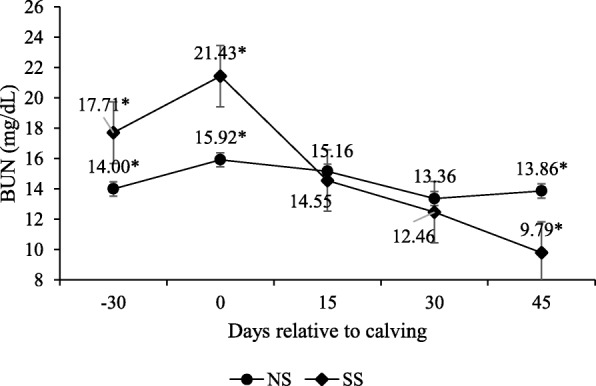
Blood urea nitrogen (BUN) concentrations during pre- and post-calving. Numbers followed by asterisks (*) are significantly different between treatments (*P* < 0.10)

An interaction occurred between supplement and peripartum days for BUN concentrations (Table 5), where concentrations were higher for the cows supplemented on day − 30 and upon calving (*P* < 0.10), and lower on 45 d post-partum (P < 0.10; Fig. 8).

**Fig. 9:**
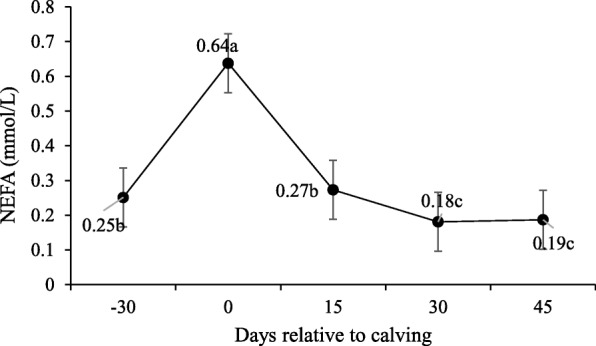
Non-esterified fatty acids (NEFA) serum concentrations during pre- and post-calving. Different letters indicate significant differences between days (*P* < 0.10)

**Fig. 10:**
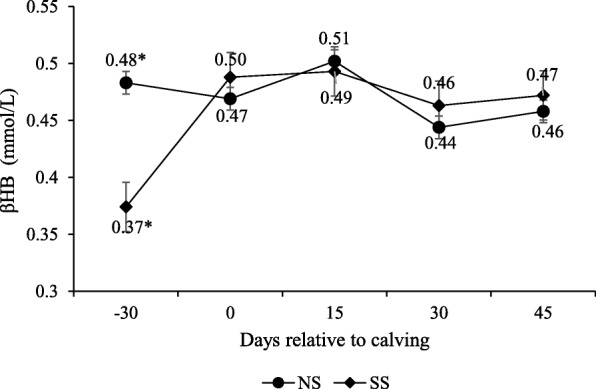
β-hydroxybutyrate (BHB) serum concentrations during pre- and post-calving. Numbers followed by asterisks (*) are significantly different between treatments (*P* < 0.10)

Serum NEFA concentrations were not affected by supplementation (*P* > 0.10; Table 5). The NEFA concentration changed along peripartum days, and its concentrations on day − 30 were lower than on the calving day and stabilized after 30 d (Fig. 9). However, an interaction was observed between supplementation and peripartum days for βHB (P < 0.10; Table 5), with the lowest concentrations on − 30-d for supplemented cows (Fig. 10).

**Fig. 11:**
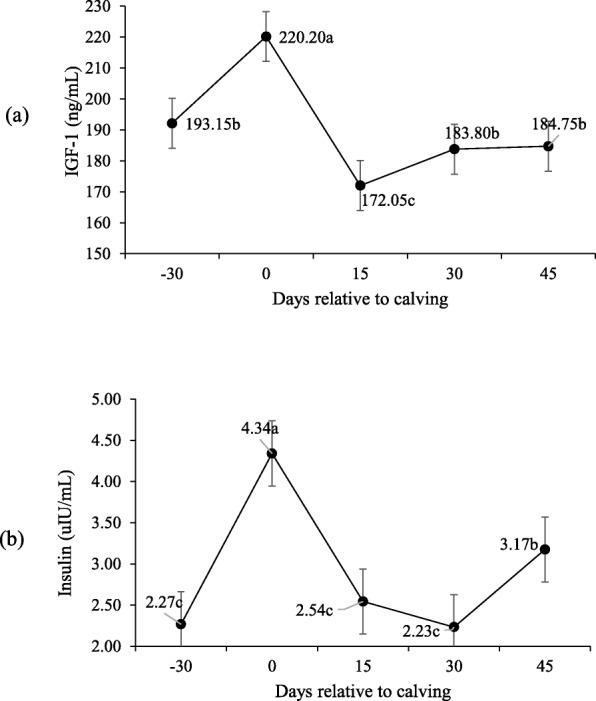
IGF-1 (**a**) e Insulin (**b**) serum concentrations during pre- and post-calving. Different letters indicate significant differences between days (*P* < 0.10)

**Fig. 12:**
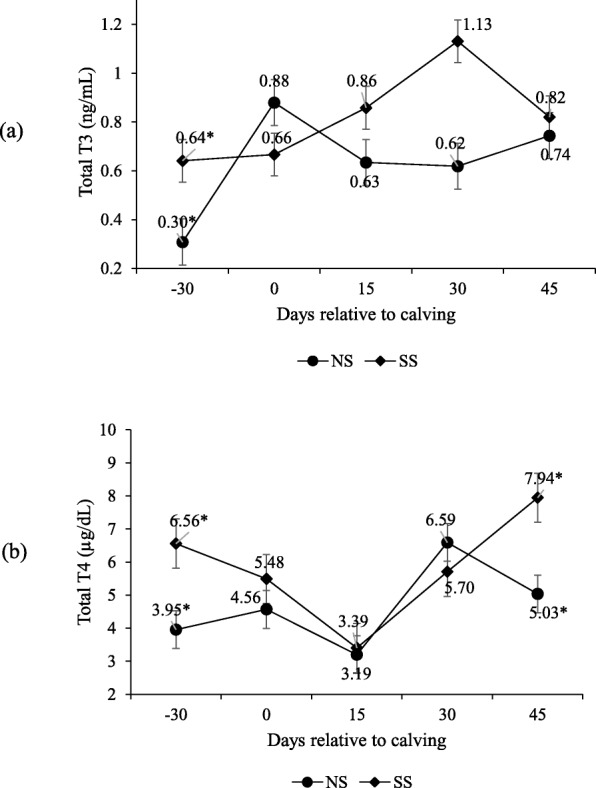
Total T3 (**a**) and T4 (**b**) serum concentrations during pre- and post-calving. Numbers followed by asterisks (*) are significantly different between treatments (*P* < 0.10)

Supplementation had no effect on insulin and IGF-1 levels, and it only changed on peripartum days (*P* > 0.10; Table 5) when both peaked upon calving (Fig. 11). An interaction occurred for both T3 and T4 between supplementation and peripartum days (P < 0.10; Table 5) when concentrations were higher on day − 30 for the supplemented cows (Fig. 12). Supplementation did not affect progesterone serum levels (Table 5), which were higher on day 45 than on day 30 (1.60 and 0.30 ng/mL).

## Discussion

Grazing cattle are usually supplemented with additional feed to increase or maintain their BCS, mainly during the dry season, or when the quality of the pasture is insufficient to meet their nutrient requirements [5, 6]. Feed supplementation of grazing cattle can lead to changes in ruminal fermentation. For example, energy-protein supplementation to cattle fed low quality forages improves ruminal fiber degradation, which leads to improved energy utilization and an increase in DM intake due to higher passage rate [11].

Findings from other studies suggest that CP levels in diet of around 100 g/kg of DM improve fiber degradation [6], and CP quantity of around 145 g/kg DM is able to improve pasture voluntary intake [12]. In this experiment, total DM intake increased due to the extra supplement feed provided to cows in the SS group but did not increase pasture intake or apNDF digestibility. Most likely, the concentration of CP supplied through the pasture and extra supplement (around 81 g/kg DM) was not high enough to have positive effects on forage intake and fiber degradation.

The supplemented cows had higher CP intake and OM and CP digestibly. This was expected because supplementation provides more dietary protein intake, apart from other highly digestible nutrients. As the NS had lower protein intake and OM digestibility, nitrogen utilization efficiency was apparently enhanced, which led to higher Emic.

During the last 60 days of gestation, cows have higher nutrient requirements [13] and, according to Silva et al. [7], supplementation administered in adequate amounts during this period can have beneficial effects on cow’s energy and protein metabolism, along with increased body reserves. Thus, it would seem to metabolically prepare cows for the postpartum period when supplements are no longer provided. Unlike Silva et al. [7], we found that pre-partum supplementation had no effect on reducing the magnitude of BW lost during the post-partum as variation in ADG was noted in the SS animals during the experimental period, which was negative during the post-partum (Fig. 1). These findings agree with Cardenas [8] and Sotelo et al. [9].

No difference in postpartum anestrus length was observed and supplementation had no effect on progesterone levels (Table 5). This indicates that supplementation was unable to contribute to greater reproductive efficiency. As expected, the above-cited results consequently reflect post-partum performance, with no difference found in pregnancy rate and days from calving to conception. These results might be explained by the fact that most of the cows presented appropriate BCS for reproduction at the beginning of the experiment (5 to 6.0 on a scale from 1 to 9) [14, 15].

According to several studies, BCS is a determining factor for cows to return to early estrus with improved conception rates [3, 16]. Furthermore, for cows with adequate BCS, there is evidence that body reserves can be used during late gestation without compromising the subsequent reproductive function [4]. This questions the need to supplement cows with adequate BCS at the end of gestation. In other words, if grazing cows are appropriately conditioned (i.e. BCS 5 to 6) towards the end of gestation and do not mobilize body reserves extensively before calving; supplementation of extra feed will not lead to improvements of the reproductive performance during the subsequent reproductive cycle.

Although pasture had low quality (less than 7 g/kg DM of CP [11]) its availability to animals sufficed and allowed selective grazing, which led NS animals to maintain ADG throughout the experimental period (Fig. 1). Therefore, no differences were found in either calves’ BW or milk production. It is well-known that BCS of cows throughout pregnancy does not impact the BW of the progeny at birth [16, 17].

The plasma glucose concentration declined regardless of the treatment at the end of gestation, which can be explained by the higher fetal demand during this period [13]. Upon calving, cows are stressed and, therefore, epinephrine acts by stimulating glycogen catabolism [18] to minimize stress during calving. Glucocorticoids act by promoting gluconeogenesis in the liver, and decrease glucose uptake and utilization in muscle and adipose tissue [19]. Hence glucose concentrations were higher during this period. The lower glucose concentrations after calving were probably caused by not only reduced DM intake, but also by higher energy demand for milk [20]. On days 30 and 45, serum glucose levels were restored and remained at basal levels.

Cholesterol levels progressively increased on all post-partum days regardless of the supplementation period. This agrees with Ruas et al. [21] and Godoy et al. [22] for postpartum blood cholesterol in lactating beef cows. Something similar occurs with HDL concentrations, which also increased after calving. In ruminants, lactogenesis increases plasma HDL concentrations, which is possibly due to an increase in either HDL synthesis or catabolism of VLDL by mammary tissue [23]. This would explain the low triglycerides and VLDL concentrations upon post-partum, and suggest its utilization as energy demand for lactation as they are important sources of fatty acids for milk fat synthesis [24]. Increased cholesterol during the postpartum could be related to precursors being needed for the synthesis of steroidal hormones [25]. While reproductive activity is re-established, avascularized granulosa cells are restricted to cholesterol uptake from HDL [26].

Data in the literature reveal that restricting pregnant cows’ intake during the late gestation period results in weight loss, BCS loss and high serum concentrations of NEFA and βHB, which lead to long periods of negative energy balance in both dairy [27, 28] and beef cows [2].

In this study, NEFA concentrations were not affected by supplementation and these levels at calving indicate a higher adipose tissue rate of lipolysis [29, 30]. The NEFA post-partum concentrations were lower compared to parturition, and remained at basal concentrations throughout the experimental period, which suggests the recovery of animals’ nutritional status. The same occurred with βHB levels upon post-partum, which differed between treatments for the pre-partum with higher concentrations for the NS animals.

Despite the difference between treatments in βHB concentrations on day − 30, these levels do not indicate intense body reserve mobilization for the NS animals, and even ADG presented differences among treatments upon the pre-partum. It is important to emphasize that most studies into energy deficit in ruminants have been done with dairy cows, and despite lack of information on beef cows serum βHB levels, it may be understood that those levels herein do not suggest severe energy deficit, rather cows had differing nutrient balances during the pre-partum.

Nevertheless, βHB levels had not influence days from calving to conception, unlike Mulliniks et al. [2] who found that low βHB concentrations were associated with an earlier conception date in beef cows. The contrasting experimental results may be explained by the fact that the βHB values that impaired reproduction cited by the above authors were higher at − 30-d (0.71 mmol/L) than those of our experiment (0.48 mmol/L). Under these conditions, the mobilization of body reserves does not lead to loss of performance and reproduction.

Albumin concentrations decreased significantly after calving, which could be related to amino acids demand for milk production [31]. Although the lowest value appeared on day 45, it still fell within the reference values (3.03–3.55 g/dL) [32]. On pre-calving day 30 and upon calving, globulin lowered in relation to the rest of the period, justified by the transfer of immunity to colostrum production [33], as reflected in the behavior of the total protein concentrations.

Unlike the other indicators of protein status, BUN levels were higher for the supplemented animals only during the pre-partum period. This is basically due to the effect of supplementation, which raised ammonia in the rumen. As urea is considered a short-time protein indicator, higher BUN levels were expected during the supplement period. Urea is synthesized in the liver in proportional amounts to the concentration of ammonia produced in the rumen, and its concentration is related directly to dietary protein levels [34].

The creatinine blood concentration is an index of muscle mass, insofar that creatinine excretion is proportional to lean body mass and is, therefore, proportional to an animal’s BW [35]. As expected, creatinine concentrations lowered linearly throughout the peripartum due to weight loss, with the lowest values on post-partum days 30 and 45, but still within the reference values (1–2 mg/dL) [32].

Feed-restricted animals display lower serum glucose and, consequently, less insulin, which reduces the somatotropin receptors in the liver, the main mediator of IGF-I production. Thus, animals in catabolic state have lower plasma IGF-I concentrations [36–38]. Therefore, IGF-I and insulin are physiologically linked and both increase with enhanced BCS. However, the regulation of each hormone individually may vary according to metabolic status, and to the direction of changes in BW.

During the experiment, insulin and IGF-1 levels behaved similarly and were not influenced by supplementation. Under similar conditions, Silva et al. [7] reported no difference in insulin levels of cows supplemented during the pre-partum. The higher values for both hormones were observed on the calving day, possibly due to blood glucose increasing and then lowering during early lactation as part of the homeorhetic changes to support galactopiesis [28]. IGF-1 concentrations were restored after 30-d post-partum, as were insulin levels on day 45, which thus stimulated steroidogenesis [39] and led to higher progesterone levels by day 45.

Food-restricted ruminants adapt to lower maintenance requirements by means of a slowing down the basal metabolism rate [40] due to lowering circulating levels of thyroid hormones. During this experiment, total T3 and T4 reduced during the pre-partum for the NS animals, which could be explained by a lowering metabolic rate compared to SS. Several works have also reported that cows during the post-partum with a negative energy balance respond to lower total T3 and T4 concentrations due to both the energy deficiency state and the huge demand of these hormones by mammary glands [41]. Conversely from parturition to 45-d, total T3 and T4 levels behave distinctly. Coggins and Field [42] demonstrated that, compared to T3, T4 serum concentrations were a more sensitive indicator of energy balance in lactating beef cows. This supports our observation of lower T4 levels on post-partum day 15, unlike T3, which did not vary much during the post-partum.

## Conclusions

Providing an energy and protein supplement to grazing Nellore cows over the last 60 d of gestation improved their pre-partum energy balance. However, no post-partum carryover effects were detected.

## Methods

### Animals, experimental design and treatments

All animal care and handling procedures were approved by the Animal Care and Use Committee of the Universidade Federal de Viçosa, Brazil (protocol CEUAP-UFV 31/17). Animals used in this study were provided by the Beef Cattle Farm of Animal Science Department at the University Federal de Viçosa, Viçosa-MG, Brazil, where the study as carried out, from July to December 2017. After the study, all cows were maintained on Beef Cattle Farm herd.

Thirty-eight pregnant multiparous Nellore cows, with an average body weight (BW) of 515 ± 11 kg, body conditions scores (BCS) of 5.5 ± 0.25 and 230 ± 10 gestation days were used. Animals were randomly divided into eight paddocks with seven hectares each, evenly covered with *Urochloa decumbens* grass, with free access to water and feeders.

The experimental design was completely randomized, with two treatments as following: NS-control; SS-cows supplemented for the 60 pre-partum days (gestation period from 230 to 290-d). The NS cows, received only a mineral mixture (MM) as loose mesh, ad libitum*,* during gestation. SS cows were group-fed with 90 kg of supplement during the pre-partum period (1.5 kg/d), accompanied by MM offered ad libitum supplied separately in additional feeders. The compositions of supplement, MM and pasture are shown in Tables 6 and 7. Treatments were randomly assigned to paddocks: six paddocks with five cows each and two with four, totalizing 19 cows per treatment. Feeders were equipped with creep-feeding and sheltered, with space of 0.3 m per cow.

**Table 6:** Ingredients and composition of supplement provided to cows at 60-d pre-partum

Item^1^	Supplement
Ingredients (%; as-fed basis)	
Corn meal	41.2
Soybean meal	36.0
Wheat meal	20.0
Urea:ammonium sulfate (9:1)	2.80
Chemical composition (g/kg of DM)	
OM	965
CP	320
apNDF	143

^1^*OM* organic matter, *CP* crude protein, *apNDF* neutral detergent fiber corrected for ash and protein residue

Mineral mix - CaHPO4 = 50.00%; NaCl = 47.775%; ZnSO4 = 1.4%; Cu2SO4 = 0.70%; CoSO4 = 0.05%; KIO3 = 0.05% and MnSO4 = 0.025%

**Table 7:** *Uruchloa decumbes* chemical composition

Item	Months			
	August^4^	September	October	November
DM^1^	651.3	762.3	505.8	236.7
OM^2^	931.8	937.6	934.1	910.4
CP^2^	48.8	52.3	58.3	82.1
apNDF^2^	749.3	770.1	731.2	592.5
iNDF^2^	338.7	347.6	362.1	177.8
NDIN^3^	217.1	190.4	259.2	425.6

*DM* Dry matter, *OM* organic matter, *CP* crude protein, *apNDF* neutral detergent fibre corrected for ash and protein, *iNDF* indigestible neutral detergent fiber, *NDIN* insoluble neutral detergent nitrogen

^1^/ g/kg of natural matter

^2^/ g/kg DM

^3^/ g/kg total nitrogen

^4^/ intake and digestibility assay

The supplement was a loose mesh formulated to contain 30% crude protein (CP) as fed to meet around 40% of CP maintenance requirements, according to BR-Corte [13]. Supplement was always provided at 11:00 h to minimize any interference of animal grazing behavior [43]. After calving, cows remained in the same paddocks, but received only MM ad libitum until 45 lactation days.

### Experimental procedures and sampling

Cows were weighed on two consecutive days at the beginning of the experiment (60-d pre-partum), and 7-d before the expected calving day to quantify the average daily gain pre-calving (ADGpre). Cows were weighed after calving and at the end of the experiment period also on two consecutive days (45-d) to quantify the average daily gain post-calving (ADGpost). Calves remained with dams during the experiment and were weighed immediately after birth, and also at 45 and 90-d. Body condition scores (BCS) were also recorded on a scale from 1 to 9, as recommended by NRC [44], by three experienced persons at the beginning of the experiment, upon calving and 45-d post-partum.

During the breeding season, starting on December 12, cows were synchronized, and fixed time artificial insemination (FTAI) was performed on December 23. Pregnancy diagnosis was made via transrectal ultrasonography 30-d after FTAI. The number of days from parturition to re-conception was calculated for each cow and pregnancy rate.

### Forage sampling

Every 30-d, grass samples were collected by hand-plucked sampling to evaluate the forage selected by animals. Samples were collected by cutting at the ground level from five delimited areas (0.5 × 0.5 m), selected randomly in each paddock to quantify DM and DMpd. In these circumstances, all the samples were weighed, oven-dried (55 °C) and then ground to pass through 1- and 2-mm screens in a Wiley mill (model 3, Arthur H. Thomas, Philadelphia, USA).

### Intake and digestibility assay

To evaluate intake and digestibility, a trial was run for 9-d on day 45 before the estimated parturition date (around 245-d of gestation). Titanium dioxide (TiO_2_) was used to estimate the fecal excretion of animals, which was wrapped in paper cartridges (20 g per animal/day) and inserted with a metal probe via the esophagus at 12:00 h [45]. The first 5 trial days were used to adapt animals to TiO_2_. Fecal samples were collected immediately after defecation or directly from the rectum on the last 4 days (one sample/day) at 18:00 h, 14:00 h, 10:00 h and 06:00 h. Feces samples were over-dried (55 °C) and ground to pass through 1- and 2-mm screens in a Wiley mill (model 3, Arthur H. Thomas, Philadelphia, USA). Then 25 g from all 4 days were pooled.

Indigestible neutral detergent fiber (iNDF) was used to estimate pasture dry matter intake (DMI) [46]. It was assumed that supplement consumption equaled the amount offered per animal/day.

On trial day 5, forage was collected by hand-plucked sampling at each paddock separately. These samples were used to estimate voluntary dry matter intake and forage digestibility.

On the trial last day, spot urine samples (5 mL) were collected 4 h before and after administering the supplement. Then a 10-mL compound was prepared with 5 mL of the urine collected in the morning and afternoon. Urine samples were diluted in 40 mL of H_2_SO_4_ (0.036 N) and then frozen (− 20 °C).

### Milk sampling

On 30- and 45-d post-calving, milking was performed to estimate milk production. In order to empty udders, calves were separated from their mothers from 15:00 h to 17:45 h, when they were reunited to dams and allowed to suckle. At 18:00 h, calves were once again separated from dams until the next morning. At 06:00 h on the next day, cows were milked immediately after an injection of 20 UI of oxytocin (10 UI/mL; Ocitovet®, Brazil) in the mammary vein and the produced milk was weighed. The exact time when each cow was milked was recorded. Calves were kept away from their mothers until the next milking at 06:00 to obtain a 24-h milk production. Next 30 mL of milk were separated from each cow to evaluate milk composition. Total production was corrected to 4% fat, according to NRC [47].

### Blood sampling

By taking calving day as day 0, blood samples were collected before feeding on days − 30, 0, 15, 30, 45. Blood samples were collected by jugular vein punctur, using vacuum tubes with a clot activator and gel for serum separation (BD Vacutainer® SST® II Advance®, São Paulo, Brazil) to quantity: blood nitrogen urea, total protein, albumin, triglycerides, total cholesterol, high density lipoprotein (HDL), nonesterified fatty acid (NEFA), beta-hydroxybutyrate (βHB), insulin, insulin-like growth factor (IGF-1), total triiodothyronine (T3), total thyroxine (T4), progesterone (P4) contents (only on 30 and 45-d). A tube with EDTA and sodium fluoride (BD Vacutainer® Fluorinated/EDTA, São Paulo, Brazil) was used to quantity the plasma glucose concentration. After collection, samples were centrifuged at 3600×g for 20 min. Serum and plasma were immediately frozen at − 20 °C until analyzed.

### Laboratory analyses

The forage, feces and supplement samples were analyzed following the procedures described by Brazilian National Institute of Science and Technology in Animal Science (INCT-CA) [48] for: dry matter (DM; index INCT-CA method G-003/1), ash (index INCT-CA method M-001/1), crude protein (CP; index INCT-CA method N-001/1), neutral detergent fiber corrected for ash and protein (apNDF; index INCT-CA method F-002/1). Indigestible neutral detergent fiber (iNDF) [49] was processed at 2 mm and quantified by in situ incubation procedures with nonwoven textile bags (100 g/m^2^) for 288 h. The fecal samples were evaluated for titanium contents using acid digestion process with concentrated sulfuric acid followed by the addition of 30% hydrogen peroxide solution and further quantification by spectrophotometry (INCT-CA method M-007/1).

With the blood samples, Bioclin® kits (Belo Horizonte, Brazil) was employed to quantity urea (K056), total protein (K031), albumin (K031), triglycerides (K117), total cholesterol (K083), HDL (K071) and glucose (K082). NEFA and βHB were analyzed using Randox® kits (FA115 and RB1007, Antrim, UK). Uric acid, creatinine and urea in urine were analyzed with kits Bioclin® (K0139, K067 and K056, Belo Horizonte, Brazil). All the above-mentioned analyses were determined by an automated biochemical analyzer (Mindray, BS200E, Shenzhen, China). Allantoin in urine was analyzed by the colorimetric method [50].

Insulin, Total T3, Total T4 and progesterone contents were analyzed by kits Beckman (33,410, 33,830, 33,800 and, 33,550 Beckman Coulter®, Brea, USA). IGF-1 contents were quantified with kits DiaSorin® (California, USA) in an automated chemiluminescence analyzer (Liaison®, Saluggia, Italy). Milk was analyzed for protein, fat, lactose and total solids content using infrared spectroscopy (Foss MilkoScan FT120, São Paulo, Brazil).

### Calculations

Potentially digestible dry matter (pdDM) was estimated using the samples collected by cutting at ground level, following the equation of Paulino et al. [51]:

$$ pdDM=0.98\ast \left(100- NDF\right)+\left( NDF- iNDF\right) $$

where 0.98 is the true digestibility coefficient of cell content; NDF is the forage content of neutral detergent fiber (%); and iNDF is the forage content of indigestible neutral detergent fiber (%).

Fecal excretion (FE) was estimated as a ratio of the TiO2 excreted in feces and the marker concentration in feces. Voluntary intake of dry matter of forage (DMF) was estimated using iNDF from forage as an internal marker following the equation of Detmann et al. [46]:

$$ \mathrm{DMF}\ \left(g/ day\right)=\left[\left( FE\ast iNDFf\right)-\left( SI\ast iNDFs\right)\right]/ iNDFfo $$

where FE = fecal excretion (kg/d), iNDFf = indigestible neutral detergent fiber in feces (kg/kg), SI = supplement DM intake (kg/d), iNDFs = indigestible neutral detergent fiber in supplement (kg/kg), and iNDFfo = indigestible neutral detergent fiber in forage (kg/kg).

The daily urine volume was estimated using the relation between daily creatinine excretion (CE) and its concentration in urine. Daily excretion was estimated by the equation according to Costa and Silva et al. [52], where shrunk body weight (SBW) was estimated as 0.88 x BW^1.0175^ [53].

$$ CE\ \left(g/ day\right)=0.0345\ast {SBW}^{0.9491} $$

Total excretion of purine derivatives was calculated by the sum of the amounts of allantoin and uric acid excreted in urine by the equation:

$$ Y=X-0.301\ast {BW}^{0.75}/0.8 $$

where Y = absorbed purine (mmol/d); X = excretion of purine derivatives (mmol/d); 0.301 = endogenous excretion of purine derivatives in urine (mmol); BW^0.75^ = metabolic weight; and 0.80 = recovery of absorbed purine as purine derivatives in urine (mmol/mmol).

Ruminal synthesis of microbial nitrogen was calculated according to the absorbed purine using the equation proposed by Barbosa et al. [54]:

$$ Z=70\ast Y/\left(0.93\ast 0.137\ast 1000\right) $$

where Z = ruminal synthesis of microbial nitrogen (g/d); Y = absorbed purine; 70 = purine N content (mg/mol); 0.93 = digestibility of microbial purine; and 0.137 = the ratio between N purine and total microbial N.

Microbial efficiency was obtained by the ratio between the production of crude microbial protein (PBmic), expressed in grams, and the amount digested organic matter intake (dMO), expressed in kilograms.

Milk production corrected to 4% fat was determined by the equation [47]:

$$ FCM=\left(0.4\ast MY\right)+\left(0.15\ast MY\ast F\right) $$

where MY = milk yield (kg/d) and F = fat yield (%).

The serum content of low-density lipoprotein (LDL) and very low-density lipoprotein (VLDL) were calculated according to Friedewald et al. [55], eq. TC = HDL + LDL + VLDL, where TC = total cholesterol and VLDL = triglycerides/5. Globulins were calculated by the difference between total proteins and albumin. Blood urea nitrogen (BUN) was estimated as 46.67% of total serum urea.

### Statistical analyses

Analyses of variance (ANOVA) for the nutritional and performance variables measured during pre- and post-calving were performed using the following model:

$$ {Y}_{ijk}=\mu +{T}_i+{e}_{(i)j}+{\varepsilon}_{(ij)k} $$

where: Y_ijk_ = observation took on animal k, within paddock, submitted to treatment I; μ = overall constant; T_i_ = fixed effect of the treatment i; e_(i)j_ = random effect of paddock j nested to treatment I, assumed to be NIID (0, σ^2^_e_); ε_(ij)_ k = random error effect associated to each observation k, which was assumed to be NIID (0, σ^2^_ε_).

The initial body weight or initial BCS were used as covariates in the model. However, when the effects of these variables on animal performance were found non-significant, the model was reparameterized excluding the covariate effect.

The measurements of ADG, metabolites and hormones were analyzed as repeated measures over time, where the best structure of (co) variance matrix was chosen based on Akaike’s information criterion with correction. Effects of treatment, day and treatment*day interaction were analyzed. When necessary, means were compared by the Fisher’s least significant difference. The pregnancy rate was evaluated using a chi-square test.

All the statistical evaluations were performed considering 0.10 as the critical level of probability for the occurrence of the type I error. In this type of experiments, animals are handled freely, hence subject to natural or unnatural disturbances, what strongly interfere to their social and intake behavior compared to feedlot experiments. Therefore, there is a higher probability of occurrence of type II error (accept H0 when is false). The best control of the type II error is obtained by increasing the α value (i.e., 0.10 rather than 0.05).

The statistical analyses were carried out using the PROC MIXED and PROC FREQ of SAS 9.4 (Inst. Inc., Cary, NC, USA).

## Data Availability

The data generated during the current study are available from the corresponding author on reasonable request.

## Abbreviations

ADGpre: Average daily gain pre-calving
ADGpost: Average daily gain post-calving
apNDF: Neutral detergent fiber corrected for ash and protein residue
BCS: Body condition score
βHB: Beta-hydroxybutyrate
BUN: Blood urea N
BW: Body weight
CP: Crude protein
DM: Dry matter
FTAI: Fixed time artificial insemination
HDL: High density lipoprotein
IGF-1: Insulin-like growth factor
iNDF: Indigestible neutral detergent fiber
MM: Mineral mixture
NDF: Neutral detergent fiber
NEFA: Nonesterified fatty acid
NS: Cows not supplemented during gestation
OM: Organic matter
P4: Progesterone
pdDM: Potentially digestible dry matter
SS: Sows supplemented for the 60 pre-partum days
T3: Total triiodothyronine
T4: Total thyroxine
